# A patient with widespread skin lesions presenting with massive pleural effusion

**DOI:** 10.5578/tt.20239610

**Published:** 2023-12-07

**Authors:** Aslıhan GÜRÜN KAYA, Pervin TOPÇUOČLU, Ayten KAYI CANGIR, Nihal KUNDAKÇI, Işınsu KUZU, Aylin OKÇU HEPER, Demet KARNAK

**Affiliations:** 1 Department of Chest Diseases, Ankara University Faculty of Medicine, Ankara, Türkiye; 2 Division of Hematology, Ankara University Faculty of Medicine, Ankara, Türkiye; 3 Department of Thoracic Surgery, Ankara University Faculty of Medicine, Ankara, Türkiye; 4 Department of Pathology, Ankara University Faculty of Medicine, Ankara, Türkiye; 5 Department of Dermatology, Ankara University Faculty of Medicine, Ankara, Türkiye

## Abstract

**ABSTRACT**

**
Latest status of non-tuberculous mycobacteria prevalence in Türkiye and the world: Systematic review
**

Non-tuberculous mycobacteria (NTM) can cause diseases not only in indivi- duals with compromised immune systems but also in those with normal immune function. This study aimed to compare the prevalence of NTM in Türkiye and worldwide between 2012 and 2022. This study was designed following the guidelines outlined in the Preferred Reporting Items for Systematic Reviews and Meta-Analyses (PRISMA) procedure. A systematic search was conducted between January 2012 and September 2022 using different electronic databases, including Pubmed, Medline, Embase, Web of Science, Ebsco, Scopus, Türk Medline, and Google Scholar. During the litera- ture review process, titles and abstracts were examined and the full texts of the studies were accessed. In 13 research articles from Türkiye included in the study, a total of 17.293 samples were studied and a total of 1304 NTM (7.54%) strains were isolated from these samples. Among the 1304 NTM strains reported from Türkiye, the top three most frequently isolated species were M. abscessus (29.83%), M. lentiflavum (14.97%), M. fortuitum (14.38%). In 35 studies included from around the world, a total of 512.626 samples were studied and a total of 12.631 NTM (2.46%) strains were isola- ted from these samples. Among the 12631 NTM strains isolated, the top three most frequently isolated species were M. intracellulare (28.13%), M. avium (17.70%) and M. abscessus (14.88%). This study unveiled the global preva- lence of NTM-infected patients, detailing species distribution and microbiolo- gical diagnostic methods. Variations in NTM spread were observed, influen- ced by diverse factors.

**Key words:** Non-tuberculous mycobacteria; prevalence; Mycobacterium abs- cessus; Mycobacterium avium

**ÖZ**

**
Türkiye ve dünyada tüberküloz dışı mikobakteri prevalansında son durum: Sistematik derleme
**

Tüberküloz dışı mikobakteriler (TDM), özellikle immün sistemi baskılanmış bireylerde hastalıklara sebep olmakla beraber, bağışıklık sistemi normal kişiler- de de hastalık oluşturmaktadır. Bu çalışmanın amacı 2012-2022 yılları arasın-

da Türkiye ve dünyadaki TDM prevalansının karşılaştırılmasıdır. Bu çalışma, Sistematik Derlemeler ve Meta Analizler için Tercih Edilen Raporlama Ögeleri (PRISMA) prosedürü kuralları baz alınarak planlanmıştır. Ocak 2012-Eylül 2022 tarihleri arasında Pubmed, Medline, Embase, Web of Science, Ebsco, Scopus, Türk Medline ve Google Scholar dahil olmak üzere farklı elektronik veri tabanları kullanarak sistematik bir tarama gerçekleştirilmiştir. Literatür tarama sürecinde başlık ve özetler incelenmiş ve çalışmaların tam metin- lerine ulaşılmıştır. Türkiye’den çalışmaya dahil edilen 13 araştırma makalesinde toplam 17,293 örnek ile çalışılmış ve bu örneklerden toplam 1304 TDM (%7,54) suşu izole edilmiştir. Türkiye’den bildirilen 1304 TDM suşu içinde en sık izole edilen ilk üç tür sırasıyla;

M. abscessus (%29,83), M. lentiflavum (%14,97), M. fortuitum (%14,38) olarak saptanmıştır. Dünya genelinden dahil edilen 35 çalışmada toplam 512,626 örnekle çalışılmış ve bu örneklerden toplam 12631 TDM (%2,46) suşu izole edilmiştir. İzole edilen 12,631 TDM suşu içinde en sık izole edilen ilk üç türün sırasıyla; M. intracellulare (%28,13), M. avium (%17,70), M. abscessus (%14,88) olduğu saptanmıştır. Bu çalışma sonucunda, dünya çapında TDM’ler ile enfekte hastaların prevalansı, türlerine göre dağılım ve mik- robiyolojik tanı yöntemleri gözler önüne sermiş olup, TDM’lerin yayılımının pek çok farklı faktöre bağlı olarak değiştiği görülmüştür.

**Anahtar kelimeler:** Tüberküloz dışı mikobakteriler; prevalans; mycobacterium abscessus; mycobacterium avium

**ABSTRACT**

**
A patient with widespread skin lesions presenting with massive pleural effusion
**

*
Mycosis fungoides is the most commonly seen type of cutaneous T-cell lymphoproliferative disease. While mycosis fungoides is linked to an increased risk of developing secondary malignancies, the occurrence of B-cell-originated disease in association with it is exceedingly rare. A 66-year-old male with persistent papillomatous skin eruption was admitted due to dyspnea. Chest X-ray, positron emission tomography, and chest com- puted tomography revealed axillary and mediastinal lymph node enlarge- ment and right lower pulmonary lobe infiltration along with right-sided massive pleural effusion. Histological and immunohistochemical findings of pleural biopsy and axillary lymph nodes suggested a diagnosis of pulmonary extranodal marginal zone lymphoma. Skin biopsies from the abdomen, chest, and legs revealed CD4/CD8 double-positive patch stage of mycosis fungoi- des. After completing six cycles of chemotherapy, complete remission of lymphoma was achieved, with the skin eruptions remaining unchanged. Herein, the authors present a unique case of concomitant diagnoses of myco- sis fungoides and marginal zone B-cell lymphoma of the respiratory system to emphasize the importance of careful evaluation of each finding.
*

**Key words:** *Mycosis fungoides; marginal zone lymphoma; pulmonary involvement; B-cell lymphoma; T-cell lymphoma
*

**ÖZ**

**
Masif plevral efüzyon ile birlikte yaygın deri lezyonları olan hasta
**

*
Mikozis fungoides, kutanöz T hücreli lenfoproliferatif hastalıkların en sık
*

*
görülen türüdür. Mikozis fungoides sekonder malignite gelişme riski ile ilişkili
*

*
olsa da B hücreli lenfomalarla birlikteliği oldukça nadirdir. Papillomatöz deri
*

*
döküntüleri olan 66 yaşında erkek hasta, nefes darlığı nedeniyle kliniğimize başvurdu. Akciğer grafisi, pozitron emisyon tomografisi ve bilgisayarlı akciğer tomografisinde aksiller ve mediastinal lenfadenopatiler, sağ alt lobda konsolidasyon ve masif plevral efüzyon görüldü. Plevra biyopsisi ve aksiller lenf nodlarının histolojik ve immünohistokimyasal bulguları pulmoner ekstranodal marjinal bölge lenfoması ile uyumlu bulundu. Karın, göğüs ve bacaklardan alınan deri biyopsileri ise CD4/CD8 pozitif mikozis fungoides ile uyumlu saptandı. Altı kür kemoterapinin ardından, marjinal zon lenfoma bulgularında belirgin gerileme saptandı. Birden fazla sistem bulgusu olan hastalarda her bir bulgunun dikkatli bir şekilde değerlendirilmesinin önemini vurgulamak için marjinal zon B hücreli lenfoma ve mikozis fungoides tanısının nadiren birlikte olduğu bir olgu sunulmaktadır.
*

**Anahtar kelimeler:** *Mikozis fungoides; marjinal zon lenfoma; pulmoner tutulum; B hücreli lenfoma; T hücreli lenfoma
*

## INTRODUCTION

Non-Hodgkin’s lymphomas are a heterogeneous group of neoplastic disorders originating from B lym- phocytes, T lymphocytes, or natural killer cells. Marginal zone B-cell lymphoma is an indolent small B-cell lymphoma originating from post-germinal center B lymphocytes (1). Primarily nodal, extranod- al, and splenic presentations are seen. Although the most common extranodal site is the gastrointestinal tract, it can arise at any extranodal site such as the lacrimal gland, salivary gland, lung, pleura, thyroid, and liver (2).

Mycosis fungoides (MF) is the most frequent type of primary skin T-cell lymphoproliferative disease with an increased risk of secondary, especially coinciden- tal existence of T-cell lymphomas (3).

Concomitant T and B-cell lymphomas are very rare. Here we report a unique case of concomitant MF with persistent papillomatous skin eruptions for 30 years and marginal zone B-cell lymphoma presenting with massive pleural effusion.

## CASE REPORT

A 56-year-old male patient was admitted to the hospital with shortness of breath, fatigue, and night

sweats. He had a history of persistent skin lesions for 30 years that gradually increased over time. Before admission, he had three interventions of thoracentesis on the right side within the last three months, with almost two liters of exudative fluid being drained each time. On physical examination of the skin, multiple indurated red-to-brown papules and plaques were seen on his chest, abdomen, back, and legs (Figure 1). Dullness on percussion and decreased breath sounds were noted over the lower third of the right hemithorax. The erythrocyte sedimentation rate and C-reactive protein level were elevated. The serum biochemistry and complete blood count were within normal ranges, as indicated in Table 1. Chest X-ray revealed massive pleural effusion and positron emission tomography with computed tomography (PET-CT scan) showed mediastinal lymphadenopathies, pleural effusion, ground-glass opacities, and consolidations (average 5-6 standardized uptake values) in middle and right lower pulmonary lobes along with peripheral such as cervical, axillary, inguinal and para-iliac lymphadenopathies (Figure 2A) (Figure 3).

Skin biopsies taken from the abdomen, chest, and legs indicated the presence of the patch stage of mycosis fungoides, characterized by a double-

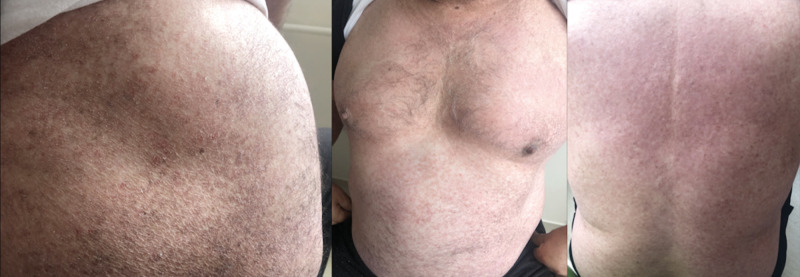

**Figure 1.** On physical examination of the skin, multiple indurated red-to-brown papules and plaques were seen.

**Table:** 

**Table 1.** Baseline laboratory findings of the patient		
**Result Reference interval**		
RBC (x1012/L)	5.41	4.2-5.6
Hemoglobin (g/dL)	15.2	13.1-17.2
Hematocrit (%)	49.3	39-50
MCV (fl)	91.2	81-101
MCH (pg/cell)	28.1	27-35
MCHC (g/dL)	30.8	32-36
RDW (%)	16	11.5-14.5
Platelets (x109/L)	269	150-400
WBC (x109/L)	8.7	4.5-11
Neutrophil (x109/L)	5.3	1.8-7.7
Lymphocytes (x109/L)	2.02	1.5-4
Monocytes (x109/L)	0.63	0.2-0.95
Eosinophils (x109/L)	0.22	0-0.7
Basophils (x109/L)	0.03	0-0.15
Glucose (mg/dL)	83	74-100
BUN (mg/dL)	11	6-20
Creatinine (mg/dL)	0.84	0.7-1.3
Na (mmol/L)	140	136-145
K (mmol/L)	4.4	3.5-5.1
LDH (U/L)	269	100-246
Uric acid (mg/dL)	9.3	3.7-8.0
Albumin (g/dL)	3.85	3.2-4.8
Beta-2 microglobulin (mg/L)	5.8	1.41-3.21
ALT (U/L)	18	10-49
AST (U/L)	10	<34
ALP (U/L)	59	46-116
GGT (U/L)	55	<73
Total bilirubin (mg/dL)	0.4	0.3-1.2
CRP (mg/L)	45.2	0-5
Procalcitonin (ng/mL)	0.02	<0.05
ESR (mm/hour)	56	<20
RBC: Red blood cell, MCV: Mean corpuscular volume, MCH: Mean cell hemoglobin, MCHC: Mean corpuscular hemoglobin concentra- tion, RDW: Red cell distribution width, WBC: White blood cell, BUN: Blood urea nitrogen, Na: Sodium, K: Potassium, LDH: Lactate dehy- drogenase, ALT: Alanine transaminase, AST: Aspartate aminotrans- ferase, ALP: Alkaline phosphatase, GGT: Gamma-glutamyl trans- ferase, CRP: C-reactive protein, ESR: Erythrocyte sedimentation rate.

positive CD4/CD8 status. Epidermotropism of atypical T lymphocytes, clusters of these cells in the epidermis (Pautrier microabscesses), or a band-like infiltrate containing abnormal lymphocytes existed

in the upper dermis (Figure 4A-G). Peripheral flow cytometry analyses revealed 35% T, 58% B, and 7% NK cells, and no Sezary cells were observed. The diagnosis of MF was established accordingly.

Flexible bronchoscopy, pleural drainage, and pleural biopsy using Abraham’s needle were performed under ultrasonic guidance. Bronchial lavage and transbronchial needle aspiration from subcarinal and right paratracheal lymph nodes were negative for malignancy and/or acid-fast bacilli (AFB), along with common bacterial and AFB cultures. A pleural biopsy was performed, involving pleuro-parenchymal tissue, which revealed diffuse infiltration of B lymphoid cells (Figure 5). These cells expressed CD20 and showed an increase in plasma cells exhibiting kappa light-chain restriction, raising suspicion of marginal zone lymphoma. Planned mediastinoscopy was cancelled due to the rapid progression of bilateral pleural fluids, including ascites and pericardial effusion. Pleural fluid flow cytometry immunophenotyping revealed 25% T, 74% B, and 1% NK cells. B-cells in pleural fluid: CD45+, HLA-DR+, CD19+, CD5-, CD20 weak+, CD10-, CD23-, FMC7-, CD43-, CD200-, CD27+,

CD49-, zap70-, kappa negative and lambda negative- comment was memory B-cell immunophenotype. AFB negative pleural fluid cytology was also compatible with the diagnosis of marginal zone B-cell lymphoma despite normal trephine biopsy. Fine needle biopsy from the axillary lymph node showed 94% lymphoid cells-comprising 59% T-cells, 40% B-cells, and 1% NK cells. The majority of B-cells tested positive for kappa. Axillary lymph node excisional biopsy examination revealed typical morphological and phenotypic characteristics of MZL involvement (Figure 6). The germinal centers were colonized by monocytoid marginal zone B-cells, in addition to interfollicular increased monotypic kappa light chain restricted plasma cells.

Combined rituximab, cyclophosphamide, vincristine, and methyl-prednisolone (R-CHOP) regimen administered (day 1: rituximab 375 mg/sqm/day,

cyclophosphamide 750 mg/sqm, vincristine 1.4 mg/ sqm and day 1-4: methyl-prednisolone 40 mg/sqm/ day for four days), may have contributed to the improvement in the skin lesions of MF. After four cycles of chemotherapy, computed tomography control revealed a remarkable resolution of mediastinal axillary, and abdominal lymph nodes as well as pleural effusion. Complete remission was

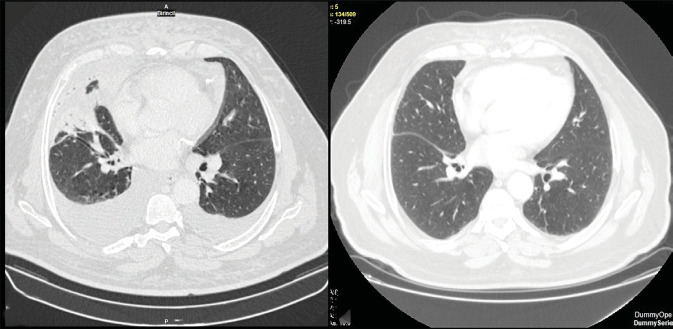

**Figure 2. (A)** Thorax computed tomography revealed pleural effusion and consolidations. **(B)**

After completing 6 cycles of chemotherapy, complete remission was obtained.

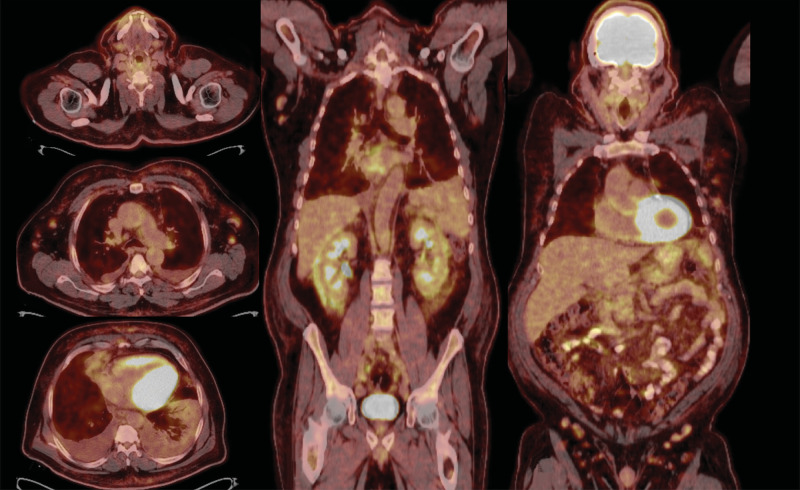

**Figure 3.** Positron emission tomography-computed tomography revealed intense fluorodeoxy-

glucose uptake in cervical, bilateral hilar, intraabdominal, inguinal lymph nodes, lung paren- chyma, and pleural fluid.

achieved after six cycles (Figure 2B). The patient’s skin lesions also mildly improved and the patient was prescribed psoralen and ultraviolet-A (PUVA) treatment. He has been doing well since then. The patient has been under follow-up for 18 months without any relapse.

## DISCUSSION

Extranodal marginal zone B-cell lymphoma is the most frequent type of primary pulmonary B-cell lymphomas, with variable clinical presentation depending upon the tissue involved. Pulmonary involvement is reported to be 10%, while pleural involvement is extremely rare (4). Either solitary or multiple parenchymal nodules, air bronchograms,

and airway dilatation are the most common radiological findings. Enlargement of mediastinal lymph nodes, pleural effusion, and pleural thickening are less commonly described (5). Cases with pleural effusion-related pulmonary marginal zone lymphoma were described in limited reports (6-8). Pleural effusion is a common finding in patients with non- Hodgkin's lymphoma; most of the diffuse large B-cell lymphoma. Pleural effusion and/or pleural thickening are also seen in primary pleural extranodal marginal zone lymphoma (9,10). Primary pleural lymphoma is a rare condition. Also, direct extension or hematogenous/lymphatic dissemination of pulmonary or nodal diseases may lead to secondary pleural involvement of non-Hodgkin's lymphoma (11).

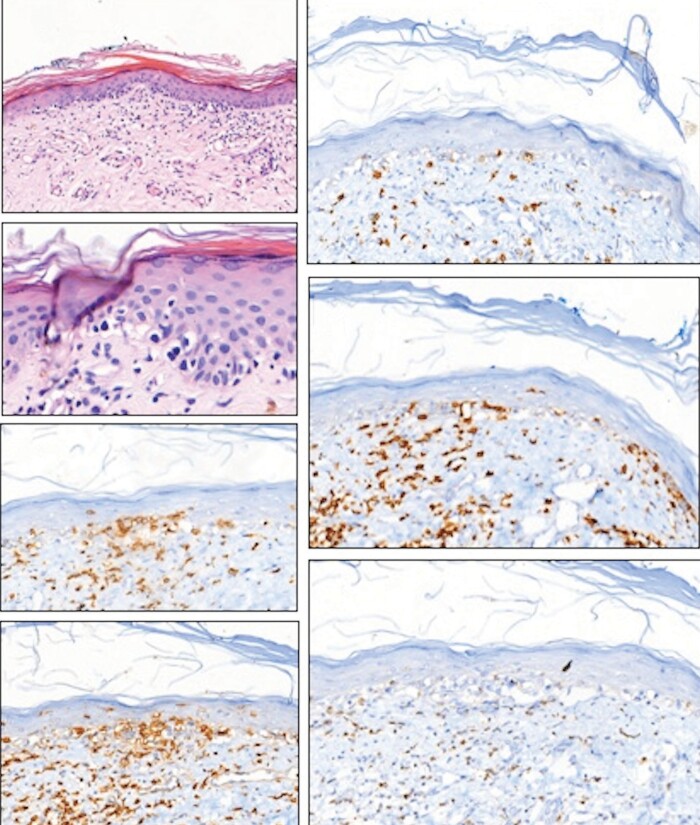

**Figure 4. (A)** Epidermotropic haloed atypical lymphocytes linearly arranged along with the basal layer of the epidermis. There is epidermal atrophy and hyperkeratosis. Dermal fibrosis and mild band-like lymphocyte infiltration with scattered melanophages are also noted. H&E x 170. **(B)** Closer view of the epidermotropic haloed atypical lymphocytes; H&E x 590. **(C)** Both haloed epidermotropic lymphocytes and dermal lymphocytes show CD3 expression; x300. **(D)** Some of the epidermotropic lymphocytes and most of the dermal lymphocytes are CD4 positive; x250. **(E)** A few of the epidermotropic lymphocytes are also CD8 positive; x170.

**(F)** Loss of CD5 expression in some of the epidermotropic T-cells; x 200 **(G)**. Loss of CD7 expression in most of the epidermotropic cells x220.

Furthermore, pleural effusion may be observed with extrinsic lymphatic or venous compression by enlarged lymph nodes (5). The presented patient had pulmonary, parenchymal, and nodal involvement shown by radiological (PET-CT), flow cytometric (pleural fluid and lymph node aspiration), and histopathological examination (pleuro-parenchymal and lymph node biopsy) revealing disseminated marginal zone lymphoma.

Although the etiology of pulmonary extranodal marginal zone lymphoma is yet not clarified, chronic immune stimulation as a result of infection or autoimmune disorder may be associated with the pathogenesis. Moreover, pulmonary extranodal marginal zone lymphoma has to be differentiated from malignancies including low-grade B-cell lymphomas and other benign lymphoproliferative disorders. The patient initially presented with progressive skin lesions diagnosed as MF, which

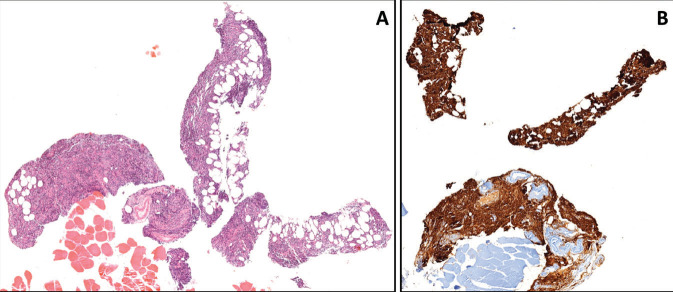

**Figure 5.** Pleuro-parenchymal biopsy revealing diffuse lymphoid infiltration **(A)** with strongly CD20 expressing atypical B-cells.

might have led to the release of autoantigens, resulting in chronic immune stimulation and the development of a secondary B-cell disease (12).

Our patient had widespread lymphadenopathy at the time of diagnosis. Generalized nodal involvement is a rare feature of extranodal marginal zone lymphoma

and predicts poor prognosis (13). So, early diagnosis and treatment yielded good results in this case, as proven previously.

MF has an association with an increased risk for the development of secondary malignancies. The coexistence of MF and B-cell malignancies in the

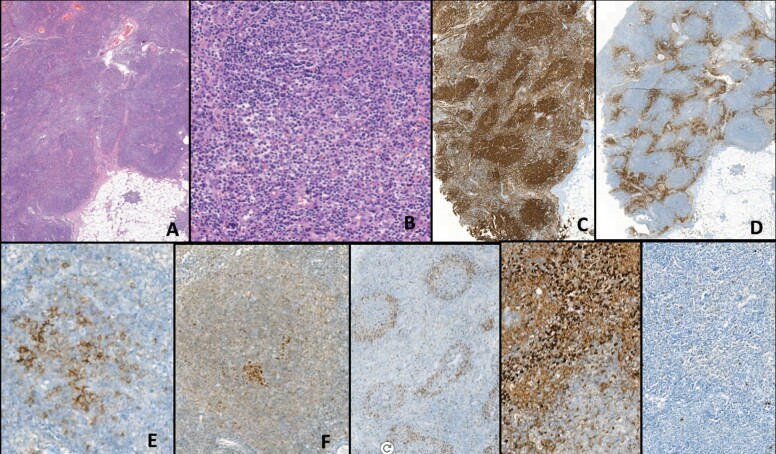

**Figure 6.** Axillary lymph node biopsy findings revealed the diagnosis of MZL involvement. The nodular infiltration by small mono-

cytoid lymphoid cells **(A)** colonizing follicles **(B)**. The colonizing lymphoid cells were CD20-expressing B-cells, and they were also increased in perifollicular areas **(C)**. CD38-expressing plasma cells were increased in interfollicular areas **(D)**. Follicular colonization, a characteristic feature of MZL, was notably evident with decreased CD23-positive follicular dendritic meshwork **(E)** and BCL6- expressing germinal center B-cells **(F)**. Ki67 proliferation index within the colonized follicles was very low, as expected **(G)**. Interfollicular increased plasma cells were clearly kappa light chain restricted **(H)** when compared with a few lambdas expressing plasma cells **(J)**.

same patient has rarely been reported (14-16). The mechanisms of secondary malignancies are unclear, although several potential mechanisms have been proposed, including the use of immunosuppressant in the treatment of the primary lesion, a genetic predisposition to malignancy, and the monoclonal proliferation of T-cells in MF, modulating the B-cell system (16,17). MF was underdiagnosed for thirty years without any treatment. His family history was also unremarkable for malignancy. Monoclonal T-cell proliferation that modulates B-cells may be a possible cause for this case as aforementioned.

Different immunophenotypic variants have been reported in MF. The neoplastic cells in MF have a mature CD4 (+), CD45RO (+), and CD8 (−) memory T-cell phenotype. In rare cases with early MF, a CD4 (−)/CD8 (+) mature T-cell phenotype or CD4/CD8 double negative immunophenotype may also be observed. CD4 (+)/CD8 (+) double-positive MF is extremely rare (18,19). We report a case of MF with a dual positive CD4/CD8 phenotype. CD4 (+)/CD8 (+) double-positivity may be the precursor of B-cell malignancy (20,21).

The differential diagnosis of MF includes mainly pseudo lymphomas, cutaneous primary B-cell lymphomas, and systemic B-cell lymphomas with cutaneous involvement. The morphological differences and the immunophenotypic characterization of neoplastic cells by immunohistochemistry and/or flow cytometry is critical to distinguish a T-cell lymphoma from a B-cell lymphoma (22,23).

Skin lesions of the patient were also evaluated in terms of B-cell lymphoma involvement because the differential diagnosis of MF includes mainly pseudo- lymphomas, cutaneous primary B-cell lymphomas, and systemic B-cell lymphomas with cutaneous involvement (16). For this reason, each patient should be evaluated carefully by biopsy via immunophenotypic characterization and immunohistochemistry, especially in patients with skin eruptions representing MF as in this case.

While systemic chemotherapies like CHOP and gemcitabine are considered treatment options for advanced-stage MF, our patient underwent CHOP therapy for B-cell lymphoma, resulting in slight regression of the skin lesions (24). PUVA treatment was postponed after the chemotherapy process because of the patient’s clinical condition.

This case report highlights a unique presentation involving widespread skin eruptions, massive pleural effusion, and the coexistence of B-cell-originated lymphoma alongside T-cell-originated MF. It underscores the importance of considering rare coexisting conditions and the necessity of a multidisciplinary approach in such cases.

## CONFLICT of INTEREST

The authors have no conflict of interest to declare.

## AUTHORSHIP CONTRIBUTIONS

Concept/Design: DK, AGK, AKC, IK Analysis/Interpretation: All of authors Writing: All of authors

Clinical Revision: All of authors Final Approval: All of authors

## REFERENCES

1. *
  Chacon JI, Mollejo M, Munoz E, Algara P, Mateo M, Lopez L, et al. Splenic marginal zone lymphoma: Clinical charac- teristics and prognostic factors in a series of 60 patients. Blood 2002; 100(5): 1648-54. https://doi.org/10.1182/ blood.V100.5.1648.h81702001648_1648_1654*
2. *
  AlShemmari SH, Ameen RM, Sajnani KP. Extranodal lym- phoma: A comparative study. Hematology 2008; 13(3): 163-9. https://doi.org/10.1179/102453308X316149*
3. *
  Berg S, Villasenor-Park J, Haun P, Kim EJ. Multidisciplinary management of mycosis fungoides/Sezary syndrome. Curr Hematol Malig R 2017; 12(3): 234-43. https://doi. org/10.1007/s11899-017-0387-9*
4. *
  Borie R, Wislez M, Antoine M, Cadranel J. Lymphoproliferative disorders of the lung. Respiration 2017; 94(2): 157-75. https://doi.org/10.1159/000477740*
5. *
  Bligh MP, Borgaonkar JN, Burrell SC, MacDonald DA, Manos D. Spectrum of CT findings in thoracic extranodal non-Hodgkin lymphoma. Radiographics 2017; 37(2): 439- 61. https://doi.org/10.1148/rg.2017160077*
6. *
  Zhao S, Zhang L, Gu Z, Zhu C, Fang S, Yang N, et al. Clinical manifestations of pulmonary mucosa-associated lymphoid tissue lymphoma: Single-center experience with 18 patients. Onco Targets Ther 2018; 11: 555-61. https:// doi.org/10.2147/OTT.S147275*
7. *
  Bachuwa G, Naik P, Campe J, Lecea N, Congdon D. Ninety-one year old: Oldest patient reported with pul- monary mucosa-associated lymphoid tissue lymphoma and rare association with pleural effusion. Geriatr Gerontol Int 2012; 12(1): 149-51. https://doi. org/10.1111/j.1447-0594.2011.00707.x*
8. *
  Arondi S, Valsecchi A, Marchetti G. Medical thoracoscopy in MALT lymphoma causing pleural effusion: A case report. Thorac Cancer 2015; 6(3): 372-4. https://doi. org/10.1111/1759-7714.12183*
9. *
  Wang X, Xie S, Ren F, Wang T, Hu X. Primary pulmonary extranodal marginal zone lymphoma of mucosa-associated lymphoid tissue with a severe hemorrhagic pleural effusion in an oldest old patient. Geriatr Gerontol Int 2017; 17(12): 2625-7. https://doi.org/10.1111/ggi.13176*
10. *
  Motta G, Conticello C, Amato G, Moschetti G, Colarossi C, Cosentino S, et al. Pleuric presentation of extranodal mar- ginal zone lymphoma of mucosa-associated lymphoid tis- sue: A case report and a review of the literature. Int J Hematol 2010; 92(2): 369-73. https://doi.org/10.1007/ s12185-010-0645-2*
11. *
  Angirish B, Sanghavi P, Jankharia B. Pulmonary manifesta- tions of lymphoma: A pictorial essay. Lung India 2020; 37(3): 263-7. https://doi.org/10.4103/lungindia.lungin- dia_200_19*
12. *
  Tan RS, Butterworth CM, McLaughlin H, Malka S, Samman PD. Mycosis fungoides-a disease of antigen persistence. Br J Dermatol 1974; 91(6): 607-16. https://doi. org/10.1111/j.1365-2133.1974.tb12449.x*
13. *
  Zucca E, Arcaini L, Buske C, Johnson PW, Ponzoni M, Raderer M, et al. Marginal zone lymphomas: ESMO Clinical Practice Guidelines for diagnosis, treatment and follow-up. Ann Oncol 2020; 31(1): 17-29. https://doi. org/10.1016/j.annonc.2019.10.010*
14. *
  Barzilai A, Trau H, David M, Feinmesser M, Bergman R, Shpiro D, et al. Mycosis fungoides associated with B-cell malignancies. Br J Dermatol 2006; 155(2): 379-86. https:// doi.org/10.1111/j.1365-2133.2006.07346.x*
15. *
  Amber KT, Bloom R, Nouri K. Second primary malignan- cies in CTCL patients from 1992 to 2011: A SEER-based, population-based study evaluating time from CTCL diagno- sis, age, sex, stage, and CD30+ subtype. Am J Clin Dermatol 2016; 17(1): 71-7. https://doi.org/10.1007/ s40257-015-0155-3*
16. *
  Miyatake J, Inoue H, Serizawa K, Morita Y, Espinoza JL, Tanaka H, et al. Synchronous occurrence of mycosis fun- goides, diffuse large B-cell lymphoma and acute myeloid leukemia. Internal Med 2018; 57(10): 1445-53. https:// doi.org/10.2169/internalmedicine.9668-17*
17. *
  Maughan C, Boudreaux L, Lear W, Bohlke A. Discordant mycosis fungoides and cutaneous B-cell lymphoma: A case report and review of the literature. JAAD Case Rep 2015; 1(4): 219-21. https://doi.org/10.1016/j. jdcr.2015.04.015*
18. *
  Tournier E, Laurent C, Thomas M, Meyer N, Viraben R, Brousset P, et al. Double-positive CD4/CD8 mycosis fun- goides: A rarely reported immunohistochemical profile. J Cutan Pathol 2014; 41(1): 58-62. https://doi.org/10.1111/ cup.12248*
19. *
  Knapp CF, Mathew R, Messina JL, Lien MH. CD4/CD8 dual-positive mycosis fungoides: A previously unrecog- nized variant. Am J Dermatopathol 2012; 34(3): e37-9. https://doi.org/10.1097/DAD.0b013e31823e25bb*
20. *
  Kaleem Z, White G, Zutter MM. Aberrant expression of T-cell-associated antigens on B-cell non-Hodgkin lympho- mas. Am J Clin Pathol 2001; 115(3): 396-403. https://doi. org/10.1309/V8YG-8PP4-B4TE-9X6J*
21. *
  Willemze R, Cerroni L, Kempf W, Berti E, Facchetti F, Swerdlow SH, et al. The 2018 update of the WHO-EORTC classification for primary cutaneous lymphomas. Blood 2019; 133(16): 1703-14. https://doi.org/10.1182/blood- 2018-11-881268*
22. *
  Dumont M, Battistella M, Ram-Wolff C, Bagot M, de Masson A. Diagnosis and treatment of primary cutaneous B-cell lymphomas: State of the art and perspectives. Cancers (Basel) 2020; 12(6): 1497. https://doi. org/10.3390/cancers12061497*
23. *
  Lima M. Cutaneous primary B-cell lymphomas: From diag- nosis to treatment. An Bras Dermatol 2015; 90(5): 687- 706. https://doi.org/10.1590/abd1806-4841.20153638*
24. *
  Photiou L, van der Weyden C, McCormack C, Miles Prince
  *

*
H. Systemic treatment options for advanced-stage mycosis fungoides and Sezary syndrome. Curr Oncol Rep 2018; 20(4): 32. https://doi.org/10.1007/s11912-018-0678-x*

